# 

**Published:** 1893-11

**Authors:** 


### CONTENTS.

ORIGINAL COMMUNICATIONS—
The Prose and Poetry of a Physician's Life. By Charles S. Webb, Ph.B., M.D., Atlanta, Ga. .... 513
Typhoid Fever. By William B. Conway, M.D., Athens, Ga. .... 521
Abstracts of Notes on the Treatment of Exophthalmic Goitre. By J. Madison Taylor, M.D., Philadelphia .... 526
Classification of Infection During the Puerperium, with Notes on Treatment. By R. R. Kime, M.D., Atlanta, Ga. .... 528
CORRESPONDENCE—
New York Letter .... 539
The Willis F. Westmoreland Memorial .... 544
EDITORIAL—
The Discovery of Modern Surgical Anesthesia .... 545
The Legislature and a Board of Examiners .... 547
The Florence Crittenton Home .... 548
SELECTIONS AND ABSTRACTS—
Skin Diseases and Syphilis .... 549
Surgery .... 555
General Medicine and Therapeutics .... 560
Practical Chemistry and Pharmacy .... 568
MEDICAL ITEMS .... 569
BOOK REVIEWS .... 572
ADVERTISERS' INDEX TO ADVERTISEMENTS .... xii
CLASSIFIED INDEX TO ADVERTISEMENTS .... xxxix
ITEMS OF INTEREST .... x, xi
PREMIUM OFFERS .... xv, xxxviii

